# Characterization of Na^+^ uptake in the endangered desert pupfish, *Cyprinodon macularius* (Baird and Girard)

**DOI:** 10.1093/conphys/cot005

**Published:** 2013-04-10

**Authors:** Kevin V. Brix, Martin Grosell

**Affiliations:** 1 Department of Biology, McMaster University, 1280 Main Street West, Hamilton, ON, Canada L8S 4K1; 2 The Rosenstiel School of Marine and Atmospheric Science, University of Miami, 4600 Rickenbacker Causeway, Miami, FL 33149, USA

**Keywords:** Carbonic anhydrase, *Cyprinodon macularius*, Na^+^–H^+^ exchanger, osmoregulation

## Abstract

This study provided an initial characterization of Na^+^ uptake in saline freshwater by the endangered pupfish, *Cyprinodon macularius.* This species occurs only in several saline water systems in the southwestern USA and northern Mexico, where salinity is largely controlled by water-management practices. Consequently, understanding the osmoregulatory capacity of this species is important for their conservation. The lower acclimation limit of *C. macularius* in freshwater was found to be 2 mM Na^+^. Fish acclimated to 2 or 7 mM Na^+^ displayed similar Na^+^ uptake kinetics, with *K*_*m*_ values of 4321 and 3672 μM and *V*_max_ values of 4771 and 3602 nmol g^−1^ h^−1^, respectively. A series of experiments using pharmacological inhibitors indicated that Na^+^ uptake in *C. macularius* was not sensitive to bumetanide, metolazone, or phenamil. These results indicate the Na^+^–K^+^–2Cl^−^ cotransporter, Na^+^–Cl^−^ cotransporter, and the Na^+^ channel–H^+^-ATPase system are likely not to be involved in Na^+^ uptake at the apical membrane of fish gill ionocytes in fish acclimated to 2 or 7 mM Na^+^. However, Na^+^ uptake was sensitive to 1 × 10^−3^ M amiloride (not 1 × 10^−4^ or 1 × 10^−5^ M), 5-(*N*-ethyl-*N*-isopropyl)-amiloride (EIPA), and ethoxzolamide. These data suggest that *C. macularius* relies on a low-affinity Na^+^–H^+^ exchanger for apical Na^+^ uptake and that H^+^ ions generated via carbonic anhydrase-mediated CO_2_ hydration are important for the function of this protein.

## Abstract (precis)

This study investigated the mechanism of Na^+^ uptake in freshwater by the endangered pupfish, *Cyprinodon macularius. Cyprinodon macularius* exhibits a low-affinity uptake system and appears to be relatively inflexibile with respect to mechanisms of Na^+^ uptake compared with most freshwater species.

## Introduction

The fish genus *Cyprinodon* (Cyprinodontiformes) is thought to have originated in the southwestern USA or northern Mexico ∼7–8 million years ago (Echelle *et al.*, 2005). Today, approximately 50 species of *Cyprinodon* have been described, with ∼40 of these species in the arid Southwest. The remaining species are spread along the Gulf of Mexico and Atlantic coastline from Venezuela to Massachusetts, as well as many of the Caribbean Islands. The majority of species live in relatively stenohaline environments (although there may be seasonal variation) ranging from slightly saline springs to athalassic lakes with salinities approximately twice that of seawater.

The desert pupfish, *Cyprinodon macularius*, historically ranged from the Gila River in Arizona to the Salton Sea in California and down into the Colorado delta region of Sonora and Baja (Miller, 1943). However, due to various water-management projects (reservoirs, water-diversion canals) and introduced species, *C. macularius* has disappeared from the Gila and lower Colorado rivers, and populations have declined at other locations (Miller and Fuiman, 1987; Dunham and Minckley, 1998). Currently, there are only two populations of *C. macularius*, one located in the Salton Sink area and the other located on the Colorado river delta.

Diversion of the Colorado river has periodically flooded the Salton Sink, forming ancient Lake Cahuilla. There have been four historical flooding events in the past 2000 years, with the lake persisting for periods of ∼50–250 years each time (Waters, 1983). The most recent diversion occurred in 1905, forming what is now called the Salton Sea, and *C. macularius* was observed relatively quickly after its formation (Thompson, 1920). The diversion ended in 1907, and salinity has been gradually increasing to its current level of ∼50 g l^−1^. Historical monitoring data on salinity in the Colorado river prior to various dam projects indicate that the feed water for the Salton Sea had an Na^+^ concentration in the range of 3–10 mM (Irelan, 1971), providing an estimate of the minimal salinity to which *C. macularius* may have been exposed in the past century. Populations of *C. macularius* are currently restricted to saline springs and creeks that drain to the Salton Sea, where salinities range from ∼1 to 50 g l^−1^ (Kinne, 1960; Martin and Saiki, 2005).

A form now recognized as *Cyprinodon eremus* occurs in Quitobaquito Springs, Arizona and in the Rio Sonoyta, Sonora, Mexico (Echelle *et al.*, 2000; Loftis *et al.*, 2009). Together, *C. macularius* and *C. eremus* are currently listed as a federally endangered species in the USA (Marsh and Sada, 1993).

Despite the impact of various man-made alterations to water flow and salinity regimens in the region, the osmoregulatory capacity of *C. macularius* has received little study. Barlow (1958) concluded that *C. macularius* had an upper salinity threshold of 90 g l^−1^ based on observations made in small hypersaline pools along the shoreline of the Salton Sea. Kinne (1960) demonstrated that larvae can survive and grow at salinities ranging from freshwater to 55 g l^−1^, with an optimal salinity between 10 and 40 g l^−1^.

Our interest was in the osmoregulatory capacity of *C. macularius* in freshwater conditions, and more specifically, in Na^+^ regulation. Freshwater teleosts must compensate for the diffusive loss of osmolytes through active uptake of ions against their chemical gradients (Evans *et al.*, 2005; Marshall and Grosell, 2006). Active Na^+^ uptake occurs primarily in gill ionocytes and is driven by Na^+^–K^+^-ATPase located on the basolateral membrane of these cells. Entry of Na^+^ from water into the cell across the apical membrane can be accomplished by several different proteins. A putativie Na^+^ channel linked to H^+^-ATPase and two Na^+^–H^+^ exchanger (NHE) isoforms are the primary mechanisms used by teleosts studied to date (Hwang *et al.*, 2011). In slightly saline waters (5–10 mM NaCl), Cl^−^-dependent Na^+^ uptake via an Na^+^–Cl^−^ cotransporter or Na^+^–K^+^–2Cl^−^ cotransporter (NKCC) has also been demonstrated in some fish (Wang *et al.*, 2009; Yang *et al.*, 2011).

We have previously characterized osmoregulation in the euryhaline coastal pupfish, *Cyprinodon variegatus variegatus*, as well as the subspecies, *Cyprinodon variegatus hubbsi* (Brix and Grosell, 2012). *Cyprinodon v. variegatus* occurs along the Gulf and Atlantic coasts of North America and tolerates salinities ranging from freshwater up to 167 g l^−1^ (Nordlie, 2006). Previous studies indicate *C. v. variegatus* does not survive (long term), grow, or reproduce in freshwater with <2 mM Na^+^ (Dunson *et al.*, 1998). In contrast, *C. v. hubbsi* occurs in only eight freshwater lakes in central Florida. These lakes have ambient Na^+^ concentrations of 0.4–1.0 mM Na^+^, below the level typically tolerated by *C. v. variegatus*, suggesting that *C. v. hubbsi* has adapted to this more dilute freshwater environment.

We demonstrated that *C. v. variegatus* and *C. v. hubbsi* bred and raised in common garden conditions (freshwater with 7 mM Na^+^) have similar low-affinity Na^+^ uptake kinetics (*K*_m_ = 7000–38 000 μM) when acclimated to 2 or 7 mM Na^+^, while *C. v. hubbsi* switches to a high-affinity system (*K*_m_ = 100–140 μM) when acclimated to low-Na^+^ freshwater (≤1 mM Na^+^) characteristic of its native habitat (Brix and Grosell, 2012). We further demonstrated, through a series of experiments with pharmacological inhibitors, that *C. v. variegatus* appears to utilize a combination of an NHE and an NKCC for Na^+^ uptake across the apical membrane of gill ionocytes at 7 mM Na^+^, but only an NHE at 2 mM Na^+^. In contrast, *C. v. hubbsi* appears to utilize only a low-affinity NHE when acclimated to 2 or 7 mM Na^+^, and a high-affinity NHE when acclimated to 0.1 or 1 mM Na^+^. Sodium uptake is not sensitive to bafilomycin (an H^+^-ATPase inhibitor) in either subspecies in any conditions, but Na^+^ uptake in *C. v. hubbsi* is sensitive to phenamil, leading to some uncertainty about the possible involvement of a Na^+^ channel–H^+^-ATPase system in this subspecies (Brix and Grosell, 2012).

Given the above findings, we hypothesized that *C. macularius*, which has evolved in relatively saline water and is rarely or never likely to encounter waters with <3 mM Na^+^, would exhibit similar Na^+^ transport kinetics and utilize the same Na^+^ transport proteins as *C. v. variegatus*. In addition to providing useful management information on this endangered species, if this hypothesis proved correct, it would provide weight of evidence regarding the basal Na^+^ transport characteristics of *Cyprinodon* and a better context in which to evaluate the apparent adaptations in *C. v. hubbsi* to osmoregulate in dilute freshwater.

## Materials and methods

### Animal holding

Adult *C. macularius* were acquired from an in-house culture maintained by the US Geological Survey laboratory in Columbia, MO (US Fish and Wildlife Service Endangered Species Permit #TE2057312). The US Geological Survey had collected their initial stock from the Salton Sea population of *C. macularius* in 2002, and the fish were maintained at 25°C in hard well water (300 mg l^−1^ hardness) adjusted to a salinity of 5 g l^−1^ with aquarium sea salts (Instant Ocean). Fish were held at the University of Miami in 110 l glass aquaria at 23–26°C in flow-through conditions, initially with filtered natural seawater (35 g l^−1^) from Bear Cut, FL, USA. Fish were acclimated to near-freshwater conditions (0.3 gl^−1^; 7 mM Na^+^, pH 7.9) over a period of 4 days and held for >30 days. After this holding period, fish were bred, and offspring were hatched and raised to sexual maturity in the same near-freshwater conditions (0.3 gl^−1^; 7 mM Na^+^, pH 7.9). Dechlorinated City of Miami tapwater (∼1.0 mM Na^+^, 1.0 mM Cl^−^, 0.5 mM Ca^2+^, 0.2 mM Mg^2+^, 0.5 mM SO_4_^2−^ and 0.8 mM HCO_3_^−^, pH 7.9) was mixed with filtered natural seawater to achieve the desired salinity. This second generation was then bred in the same conditions to produce fish used for all experiments in this study. Throughout the acclimation and holding period, as well as during all experiments, *C. macularius* were fed *Artemia* nauplii for 2 weeks posthatch and then, over a 1 week period, gradually switched over to flake food (Tetramin™ Tropical Flakes).

### Characterization of Na^+^ uptake kinetics

The Na^+^ uptake kinetics of *C. macularius* were determined in juvenile fish (26–240 mg) acclimated to 2 or 7 mM Na^+^ for at least 3 weeks prior to experimentation. In a preliminary experiment, attempts to acclimate juvenile and adult *C. macularius* to 1 mM Na^+^ over a 4 day period resulted in ∼50% mortality within 96 h, so 2 mM Na^+^ was considered the lower limit for this species. For each experiment, Na^+^ uptake rates were measured at seven or eight different ambient Na^+^ concentrations ranging from 0.174 to 57.1 mM Na^+^ depending on the Na^+^ concentration to which they were acclimated. At each Na^+^ concentration, eight juvenile fish were placed in 50 ml of a defined medium (480 μM CaSO_4_, 150 μM MgSO_4_ and 100 μM KHCO_3_, pH 7.0, 23°C), to which a targeted concentration of NaCl was added. Test solutions were continuously aerated to maintain dissolved oxygen levels during the flux period. Fish were allowed to acclimate to this medium for 10 min, after which the medium was replaced with fresh solution of the same ionic composition, and 1–2 μCi of ^22^Na (depending on ambient Na^+^ concentration) was added to the solution. The flux solution (1 ml) was sampled after 1 min for measurements of [Na^+^] and ^22^Na activity. The total flux exposure period ranged from 0.9 to 2.3 h, depending on the ambient Na^+^ concentration being tested. In all cases, the internal specific activity was <1% of the external specific activity (assuming 150 mM Na^+^ in plasma) such that correction for backflux was unnecessary (Maetz, 1956). At the end of the exposure period, water samples for [Na^+^] and ^22^Na activity were again collected, fish were removed from the exposure media, double rinsed in a 100 mM Na^+^ solution to displace any loosely bound ^22^Na, blotted dry, euthanized with an overdose of MS-222, weighed to nearest 0.1 mg, and then assayed individually for radioactivity.

### Pharmacological inhibitor experiments

Juvenile *C. macularius* were acclimated to different Na^+^ concentrations as described above. Experiments were then performed to measure Na^+^ uptake in the presence and absence of different pharmacological inhibitors. Initial experiments were conducted using amiloride (*N*-amidino-3.5-diamino-6-chloropyrazinecarbromide), which inhibits both Na^+^ channels and NHEs, with a higher affinity for Na^+^ channels (Kleyman and Cragoe, 1988). We tested three amiloride concentrations in an attempt to distinguish effects between the Na^+^ channel and NHE pathways. For the control and amiloride treatments, eight juvenile *C. macularius* were exposed in 48 ml of the water to which they were acclimated. Fish were allowed to acclimate for 10 min to the test system, after which the water was replaced with fresh solution of the same ionic composition. Amiloride dissolved in dimethyl sulfoxide (DMSO) was then added to achieve final concentrations of 1 × 10^−5^, 1 × 10^−4^, or 1 × 10^−3^ M amiloride and 0.1% DMSO, while for the control group only DMSO was added. After allowing 5 min for the drug to take effect, 0.2 μCi of ^22^Na was added to each treatment, and the fish were exposed for 2 h. At the beginning and end of the exposure period, a 1 ml sample was collected for measurement of [Na^+^] and ^22^Na activity. At the end of the exposure period, fish were treated as described in the Na^+^ uptake experiments above.

Similar experimental designs were used in subsequent inhibitor experiments, again testing fish acclimated to 2 or 7 mM Na^+^. *Cyprinodon macularius* were exposed to 5 × 10^−5^ M 5-(*N*-ethyl-*N*-isopropyl)-amiloride (EIPA), which is a potent NHE inhibitor with low affinity for Na^+^ channels, and 1 × 10^−5^ M phenamil, a Na^+^ channel inhibitor with relatively low affinity for NHEs (Kleyman and Cragoe, 1988). In order to investigate the presence/absence of a chloride-dependent Na^+^ transporter, juvenile fish were exposed to 1 × 10^−4^ M bumetanide (NKCC inhibitor) and 1 × 10^−5^ M metolazone (Na^+^–Cl^−^ cotransporter inhibitor) in separate experiments. Finally, we investigated the potential role of carbonic anhydrase (CA) in Na^+^ uptake by exposing fish to 10^−4^ M ethoxzolamide (6-ethoxy-1,3-benzothiazole-2-sulfonamide), a potent CA inhibitor.

### Analytical methods, calculations, and statistical analysis

Total Na^+^ in water samples was measured by atomic absorption spectrophotometry (Varian Spectra AA-220, Mulgrave, Victoria, Australia). Water and fish samples were measured for ^22^Na activity using a γ-counter with a window of 15–2000 keV (Packard Cobra II Auto-Gamma, Meriden, CT, USA). Rates of Na^+^ uptake as measured by the appearance of radioactivity in the fish (in nanomoles per gram per hour) were calculated following Boisen *et al.* (2003).

All values are expressed as means ± SEM. Comparison data were analysed by Student's unpaired *t*-test (or Mann–Whitney *U*-test if normality assumptions were not met) or by ANOVA with a *post hoc* Holm–Sidak test when multiple treatments were evaluated. All comparison analyses were performed using SigmaStat v3.5 (SPSS, 2006). Kinetic data were observed to fit a Michaelis–Menten function, and estimates of *K*_m_ and *V*_max_ were determined using non-linear regression in GraphPad Prism v5.0 (GraphPad Software Inc., 2007). Differences in *K*_m_ and *V*_max_ estimates for fish acclimated to different Na^+^ concentrations were tested using an extra sum of squares *F*-test (Zar, 2009).

## Results

### Sodium uptake kinetics

**Figure 1:**
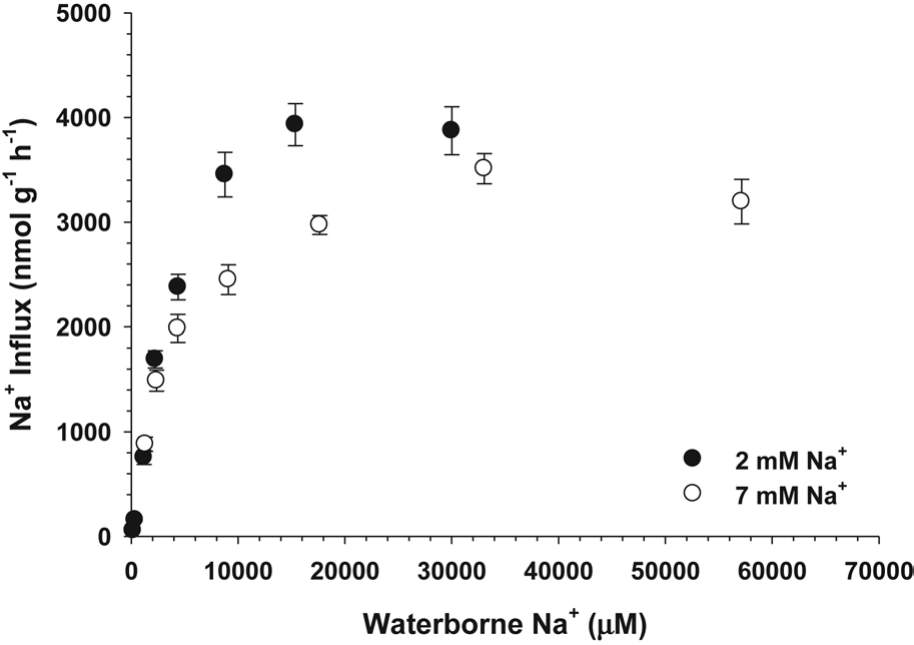
Na^+^ uptake rates (in nanomoles per gram per hour) as a function of external Na^+^ concentrations (micromolar) for *Cyprinodon macularius* acclimated to 2 or 7 mM Na^+^. Values are means ± SEM (n* *= 8). See Table 1 for estimates of *K*_m_ and *V*_max_

Sodium uptake rates increased with increasing ambient Na^+^ concentrations and followed a hyperbolic curve that approximated Michaelis–Menten saturation kinetics for *C. macularius* acclimated to both 2 and 7 mM Na^+^ (Fig. 1). The apparent *K*_m_ values were statistically similar between the two salinities (*F*_1,116_ = 0.888, *P* = 0.348), while the estimated *V*_max_ for fish acclimated to 2 mM Na^+^ was significantly higher (*F*_1,116_ = 31.09, *P* < 0.0001) than observed for fish acclimated to 7 mM Na^+^ (Table 1).

**Table 1.** Na^+^ uptake kinetics in *Cyprinodon* spp.

Organism	Size (g)	Acclimation water [Na^+^] (μM)	*K*_m_ (μM)	*V*_max_ (nmol g^−1^ h^−1^)	Reference
*Cyprinodon macularius*	0.03	2000	4321 ± 843^a^	4771 ± 303^a^	Present study
	0.10	7000	3672 ± 540^a^	3602 ± 138^b^	Present study
*Cyprinodon variegatus hubbsi*	0.12	100	104 ± 14^b^	5232 ± 234^a^	Brix and Grosell (2012)
	0.30	1200	110 ± 52^b^	1437 ± 193^c^	Brix and Grosell (2012)
	0.23	2000	7464 ± 1615^c^	10 878 ± 904^d^	Brix and Grosell (2012)
	0.31	7000	6975 ± 996^c^	6370 ± 348^e^	Brix and Grosell (2012)
*Cyprinodon variegatus variegatus*	0.13	1000	2027 ± 175^d^	8640 ± 360^f^	Brix and Grosell (2013)
	0.42	2000	18 509 ± 3342^e^	18 999 ± 1560^g^	Brix and Grosell (2012)
	0.53	7000	38 271 ± 8321^f^	30 681 ± 3393^h^	Brix and Grosell (2012)

Different letters indicate statistically significant differences (*P* < 0.05).

### Pharmacological inhibitor experiments

**Figure 2:**
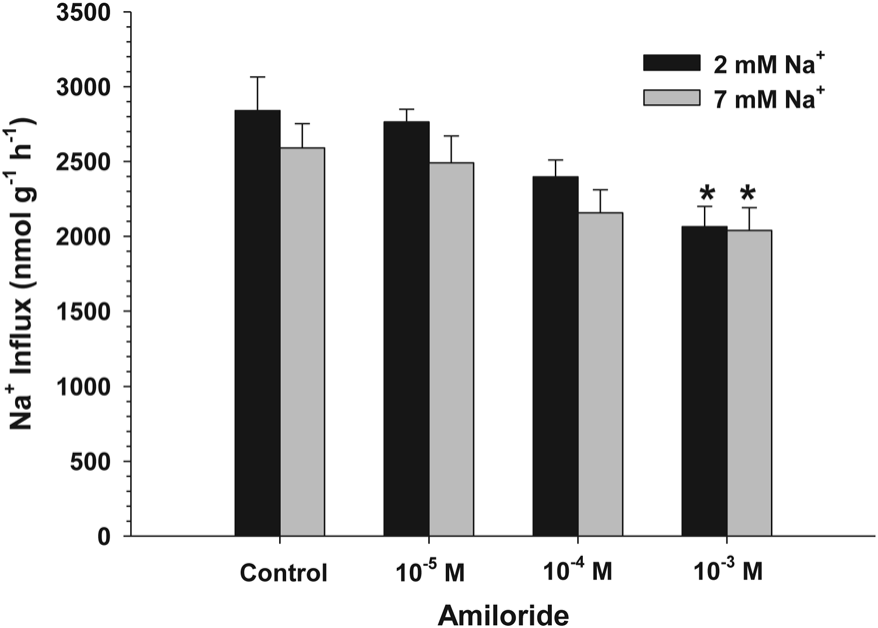
Effect of increasing amiloride concentrations on Na^+^ uptake rates (in nanomoles per gram per hour) in *C. macularius* acclimated to 2 or 7 mM Na^+^. Controls include dimethyl sufoxide (DMSO) carrier. Values are shown as means + SEM (*n* =* *8). *Statistical difference compared with the control (*P* ≤ 0.05)

**Figure 3:**
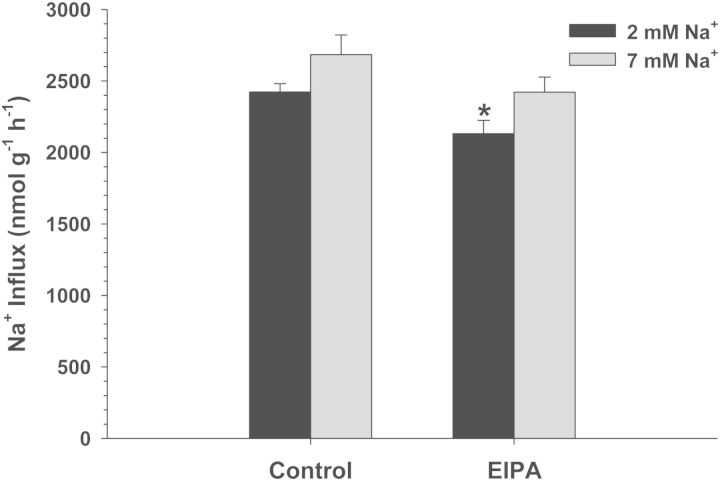
Effect of 5 × 10^−5^ M 5-(*N*-ethyl-*N*-isopropyl)-amiloride (EIPA) on Na^+^ uptake rates (in nanomoles per gram per hour) in *C. macularius* acclimated to 2 or 7 mM Na^+^. Controls include DMSO carrier. Values are shown as means + SEM (*n*** **=** **8). *Statistical difference compared with the control (*P* ≤ 0.05)

**Figure 4:**
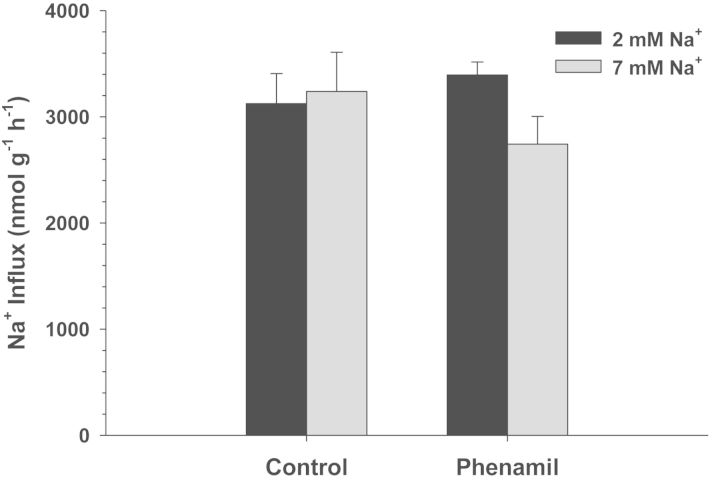
Effects 1 × 10^−5^ M phenamil on Na^+^ uptake rates (in nanomoles per gram per hour) in *C. macularius* acclimated to 2 or 7 mM Na^+^. Controls include DMSO carrier. Values are shown as means + SEM (*n*** **=** **8).

**Figure 5:**
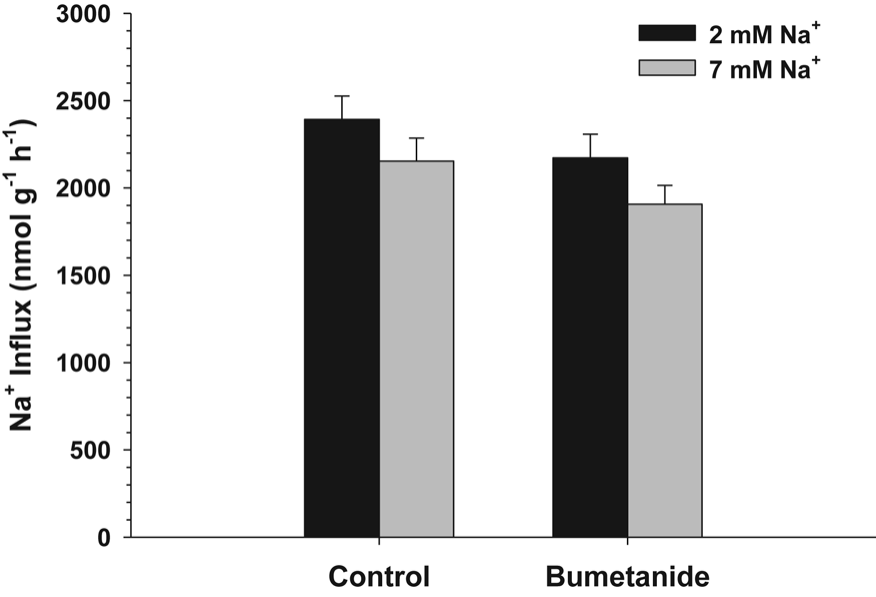
Effect of 1 × 10^−4^ M bumetanide on Na^+^ uptake rates (in nanomoles per gram per hour) in *C. macularius* acclimated to 2 or 7 mM Na^+^. Controls include DMSO carrier. Values are shown as means + SEM (*n*** **=** **8).

**Figure 6:**
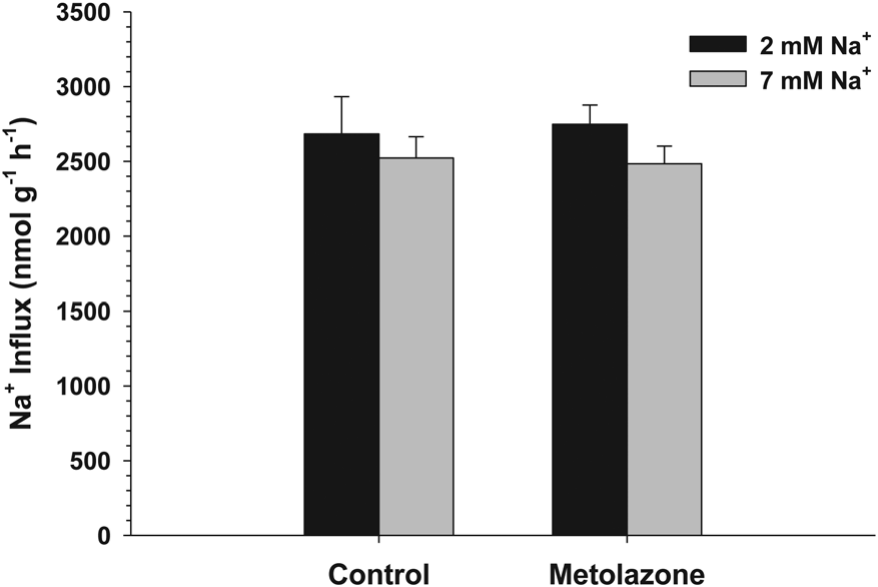
Effect of 1 × 10^−5^ M metolazone on Na^+^ uptake rates (in nanomoles per gram per hour) in *C. macularius* acclimated to 2 or 7 mM Na^+^. Controls include DMSO carrier. Values are shown as means + SEM (*n*** **=** **8).

**Figure 7:**
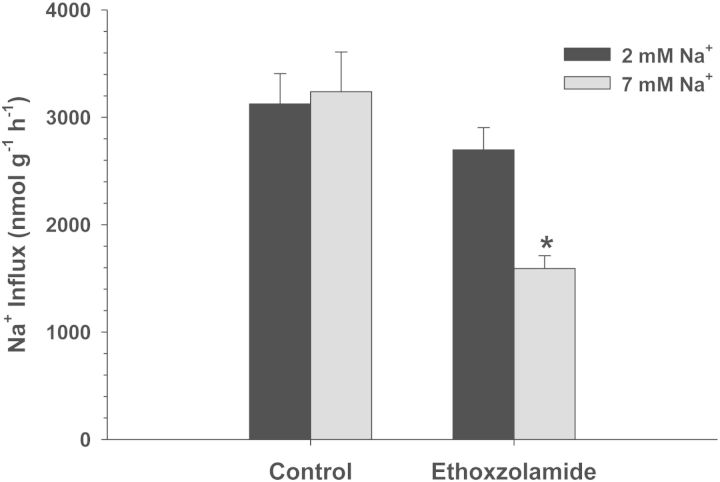
Effect of 1 × 10^−4^ M ethoxzolamide on Na^+^ uptake rates (in nanomoles per gram per hour) in *C. macularius* acclimated to 2 or 7 mM Na^+^. Controls include DMSO carrier. Values are shown as means + SEM (n = 8). *Statistical difference compared with the control (P ≤ 0.05)

Exposure of *C. macularius* acclimated to 2 and 7 mM Na^+^ to increasing concentrations of amiloride resulted in progressively increasing inhibition of Na^+^ uptake (Fig. 2). However, even at the highest amiloride concentrations tested (1 × 10^−3^ M), Na^+^ uptake was inhibited by only 22–27%, precluding any estimates of *K*_0.5_. The NHE-specific inhibitor EIPA also had a very modest effect, and only in the 2 mM Na^+^ acclimated fish (*t*_13_ = 2.738, *P* = 0.017; Fig. 3). No significant inhibition of Na^+^ uptake was observed at either salinity in experiments with phenamil, bumetanide, and metolazone (Figs 4–6). The final experiment with the CA inhibitor, ethoxzolamide, resulted in 14 and 51% inhibition of Na^+^ uptake in 2 and 7 mM Na^+^ acclimated fish, respectively, with the latter being significantly different from the control (Mann–Whitney *U* = 3.00, *n* = 7, *P* = 0.002; Fig. 7).

## Discussion

Our objective was to assess Na^+^ transport characteristics in *C. macularius* and compare them with those previously observed in *C. v. variegatus* and *C. v. hubbsi*. We hypothesized that *C. macularius* would be similar to *C. v. variegatus*, with previously observed differences between *C. v. variegatus* and *C. v. hubbsi* reflecting adaptations by the latter to its dilute freshwater environment.

With respect to Na^+^ transport kinetics, *C. macularius* displayed a significantly lower *K*_m_ than either *C. variegatus* subspecies when acclimated to 7 mM Na^+^, but was intermediate when acclimated to 2 mM Na^+^ (Table 1). This change in ranking reflects the lack of change in *C. macularius K*_m_ over this range, while both *C. variegatus* subspecies significantly increased their affinity for Na^+^ with decreasing ambient Na^+^ concentrations. The lack of flexibility in apparent *K*_m_ for *C. macularius* may reflect the relatively stenohaline environment (i.e. not subject to daily fluctuations in salinity due to tidal cycles) in which it has evolved, in comparison to *C. variegatus*.

Similar to *C. v. variegatus*, lack of phenamil sensitivity in *C. macularius* (Fig. 4) indicates that they do not utilize the apical Na^+^ channel–H^+^-ATPase system that is used in the majority of freshwater fish studied to date (Hwang *et al.*, 2011). Interestingly, the only other species studied to date that lack this system are *Fundulus heteroclitus* (Patrick and Wood, 1999; Scott *et al.*, 2005) and the Japanese medaka (*Oryzias latipes*; Wu *et al.*, 2010). *Fundulus heteroclitus* is in the order Cyprinodontiformes, while *O. latipes* is in the closely related order Beloniformes (Parenti, 2008). Both orders include a large number of small euryhaline fish, suggesting a possible phylogenetic and/or environmental signal for this difference in Na^+^ transport protein expression. It would be of interest to study a euryhaline fish outside of these two orders to test whether the absence of the apical Na^+^ channel–H^+^-ATPase system is characteristic of these two orders or a trait common to euryhaline fish.

*Cyprinodon macularius* Na^+^ uptake was also insensitive to both bumetanide and metolazone (Figs 5 and 6), indicating that this species does not use either NKCC or the Na^+^–Cl^−^ cotransporter for apical Na^+^ acquisition. We previously hypothesized that the very high *V*_max_ for *C. v. variegatus* was primarily due to the expression of NKCC in the apical membrane of gill ionocytes in this species (Brix and Grosell, 2012). The apparent lack of NKCC expression in *C. macularius* and correspondingly low *V*_max_, provides further support for this hypothesis. We also hypothesized that the expression of an apical NKCC in *C. v. variegatus* ionocytes was an evolved trait not basal to *Cyprinodon* spp. and may be an adaptation to take up large amounts of Na^+^ rapidly when it occurs high in estuaries, where >1 mM Na^+^ concentrations may occur for only a few hours during high tide. The lack of NKCC in the more stenohaline *C. macularius*, as well as the loss of NKCC expression from the stenohaline *C. v. hubbsi* (Brix and Grosell, 2012), is consistent with this hypothesis.

*Cyprinodon macularius* Na^+^ uptake was sensitive to both amiloride, a Na^+^ channel and NHE blocker, and EIPA, a NHE specific inhibitor. Amiloride typically affects Na^+^ channels at relatively low concentrations (10^−5^ M), while inhibiting NHE at higher concentrations (10^−4^ to 10^−3^ M; Kleyman and Cragoe, 1988). Considering that Na^+^ uptake is phenamil insensitive, that only 1 × 10^−3^ M amiloride inhibited Na^+^ uptake (Fig. 2), and that EIPA had a comparable effect to this amiloride concentration (Fig. 3), it appears that *C. macularius* is relying on a relatively low-affinity NHE for apical Na^+^ uptake. However, it is worth noting that the *K*_0.5_ for *C. macularius* was >1 × 10^−3^ M amiloride, significantly higher than that estimated for *C. v. variegatus* (3 × 10^−4^ M). Likewise, EIPA inhibited Na^+^ uptake only by 12% in *C. macularius* acclimated to 2 mM Na^+^, while it inhibited Na^+^ uptake by 51 and 91% in *C. v. variegatus* and *C. v. hubbsi* acclimated to the same water (Brix and Grosell, 2012). This raises the possibility that *C. macularius* is using a different NHE isoform, that there are amino acid differences for the same protein isoform, or that other currently unidentified proteins are involved in Na^+^ uptake across the apical membrane of gill ionocytes.

The final experiment involved use of the CA inhibitor, ethoxzolamide. In some fish, CA-mediated hydration of CO_2_ provides intracellular H^+^ for the function of NHE in thermodynamically unfavourable conditions (Hirata *et al.*, 2003). We hypothesized that inhibition of CA would have little or no effect on Na^+^ uptake at 7 mM Na^+^, because this condition is favourable for NHE function, but would potentially affect Na^+^ uptake at 2 mM Na^+^, where the Na^+^ gradient would be less thermodynamically favourable. Surprisingly, we observed that ethoxzolamide did not have a significant effect on Na^+^ uptake at 2 mM Na^+^, but did have a significant effect at 7 mM Na^+^ (Fig. 7). These results contrast with *C. v. variegatus*, in which treatment with ethoxzolamide stimulated Na^+^ uptake at 7 mM Na^+^, but inhibited Na^+^ uptake at 2 mM Na^+^. The present results for *C. macularius* are more similar to *C. v. hubbsi*, in which ethoxzolamide inhibited Na^+^ uptake at both 2 and 7 mM Na^+^, albeit to a lesser extent than observed for *C. macularius* (Brix and Grosell, 2012).

We previously hypothesized that the stimulatory effect observed in *C. v. variegatus* was the result of an unknown signalling pathway activating NKCC, and the lack of stimulation in *C. macularius* is consistent with this hypothesis. The reason for the greater effect of ethoxzolamide in *C. macularius* at 7 mM Na^+^ compared with 2 mM Na^+^ is not clear. It suggests that the thermodynamic gradient is less favourable at 7 mM Na^+^. There are several possible reasons for this; intracellular Na^+^ may be higher in fish acclimated to 7 mM Na^+^, requiring a higher production of H^+^ by CA to maintain a favourable gradient, or an NHE-Rhesus glycoprotein metabolon (Wu *et al.*, 2010; Kumai and Perry, 2011) may be active only in fish acclimated to 2 mM Na^+^, which would be likely to dampen the effects of ethoxzolamide on Na^+^ uptake. One possibility worth investigating is that *C. macularius* switches Na^+^–K^+^-ATPase isoforms (Jorgensen, 2008; Liao *et al.*, 2009) between 7 and 2 mM Na^+^, allowing for a lower intracellular Na^+^ and therefore less reliance on CA-generated H^+^ for NHE function. Regardless of the mechanism, the results for *C. macularius* and the two *C. variegatus* subspecies highlight the diverse and complex interplay of CA with proteins involved in Na^+^ uptake in freshwater, even among closely related species.

In conclusion, this study suggests that the endangered pupfish, *C. macularius*, may rely solely on a low-affinity NHE for apical Na^+^ uptake in freshwater, although we cannot rule out the possibility of novel transport systems being present, given the modest effects observed in the inhibitor experiments. This contrasts with two subspecies of *C. variegatus* that display a more dynamic response to varying Na^+^ concentrations in freshwater, utilizing either a combination of NKCC and NHE or multiple NHE isoforms for apical Na^+^ uptake. Similar to *C. variegatus*, H^+^ generated by CA-mediated hydration of CO_2_ plays an important role in regulating Na^+^ uptake by *C. macularius* in freshwater.
